# An adult patient with Henoch-Schönlein purpura and non-occlusive mesenteric ischemia

**DOI:** 10.1186/1756-0500-6-26

**Published:** 2013-01-23

**Authors:** Chiyako Oshikata, Naomi Tsurikisawa, Masakazu Takigawa, Tomoko Omori, Satoshi Sugano, Takahiro Tsuburai, Hiroyuki Mitomi, Tamiko Takemura, Kazuo Akiyama

**Affiliations:** 1 Department of Allergy and Respirology, National Hospital Organization Sagamihara National Hospital, 18-1 Sakuradai, Minami-ku, Sagamihara, Kanagawa 252-0392, Japan; 2 Department of Radiology, National Hospital Organization Sagamihara National Hospital, 18-1 Sakuradai, Minami-ku, Sagamihara, Kanagawa 252-0392, Japan; 3 Department of Gastroenterology, National Hospital Organization Sagamihara National Hospital, 18-1 Sakuradai, Minami-ku, Sagamihara, Kanagawa 252-0392, Japan; 4 Department of Human Pathology, Juntendo University, 2-1-1 Hongo, Bunkyo-ku, Tokyo, 113-8421, Japan; 5 Department of Pathology, Japanese Red Cross Medical Center, 4-1-22 Hiroo, Shibuya-ku, Tokyo, 150-8935, Japan

**Keywords:** Henoch-Schönlein purpura, Intervention, Non-occlusive mesenteric ischemia, Small vessel vasculitis

## Abstract

**Background:**

Onset of Henoch-Schönlein purpura (HSP) in middle age is uncommon, and adults with renal or gastrointestinal involvement present with more severe disease than do similar pediatric patients.

**Case presentation:**

We present the case of a 69-year-old male with HSP who, after treatment with steroids, cyclophosphamide, and continuous intravenous prostaglandin E1 (PGE1), died as a result of severe gastrointestinal involvement with non-occlusive mesenteric ischemia (NOMI). Vascular narrowing associated with the NOMI improved after catheter injection of PGE1 and prednisolone, but the patient died of bleeding from an exposed small vessel. At autopsy there was no active vasculitis in the jejunal submucosa.

**Conclusion:**

Treatment with PGE1 and prednisolone might improve small-vessel vasculitis associated with NOMI.

## Background

Henoch-Schönlein purpura (HSP) is characterized by a leukocytoclastic vasculitis involving the small vessels, with deposition of immune complexes that contain IgA [1,2]. Clinical signs include purpura, arthralgia, glomerulonephritis, and gastrointestinal involvement [3]. HSP occurs primarily in children [4]. It is uncommon in people over the age of 40, and little is known about its natural history in this population [5]. The prognosis for patients with childhood-onset HSP is good. However, the clinical presentation of HSP in adults is severe and the clinical outcome relatively poor [6]. In particular, deaths have been reported in cases of adult HSP with severe gastrointestinal involvement [7,8].

Non-occlusive mesenteric ischemia (NOMI) is defined as acute mesenteric ischemia through hypoperfusion caused by ongoing splanchnic vasoconstriction, without demonstrable occlusion of the mesenteric vasculature [9]. NOMI is a life-threatening vascular emergency that requires early diagnosis and intervention to adequately restore mesenteric blood flow and prevent bowel necrosis and patient death. Risk factors for NOMI include hypovolemia, hypotension, low cardiac output status, renal or hepatic disease, cardiac surgery, and administration of α-adrenergic agonists, digoxin, or β-receptor blocking agents [9]. There are no published reports of NOMI associated with HSP.

We present a fatal case of HSP in an adult patient who had severe gastrointestinal involvement with NOMI. After treatment with steroids, cyclophosphamide, intravenous steroids, prostaglandin E1 (PGE1), and continuous intravenous papaverine hydrochloride, the ischemic change caused by small-vessel vasculitis of the small intestine improved. Unfortunately, the patient had a poor prognosis owing to bleeding from an exposed small vessel, although at autopsy he had no apparent active vasculitis in the jejunal submucosa.

## Case presentation

A 69-year-old Japanese male presented with a history of bronchial asthma from age 61 and hypertension from age 50. He had been treated with inhaled glucocorticosteroids, long-acting inhaled β2-agonists, leukotriene modifiers, methylxanthines, and antihypertensives. He was an ex-smoker with a Brinkman Index of 1200. He had noticed purpura appearing in both lower extremities without any preceding infection, including upper respiratory tract infection. He then developed, without fever, edema in both lower extremities and arthralgia in the right foot. Four days after the patient had first noticed symptoms, the purpura gradually improved. However, he began suffering from abdominal pain, diarrhea, edema of the upper extremities, and oliguria.

**Figure 1:**
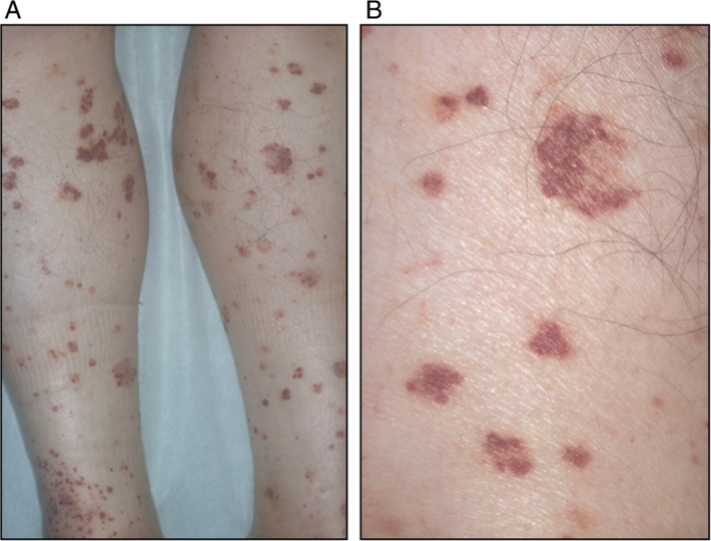
**Purpura. **(**A**) Diffuse small palpable purpura on the lower extremities. (**B**) Magnified view.

**Figure 2:**
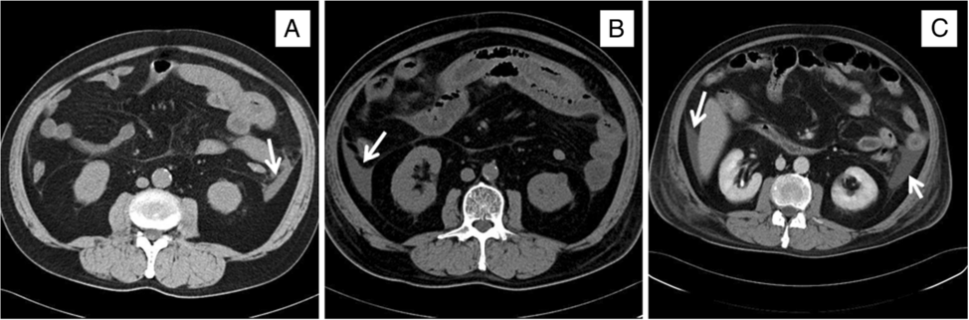
**Computed tomography of the abdomen. **(**A**) Computed tomography of the abdomen performed on admission, showing diffuse thickening of the bowel wall with target signs in the small intestine and ascites (arrow). (**B**) Computed tomography of the abdomen performed on day 9, showing severe edematous and dilatational changes in the small intestine and ascites (arrow) before treatment with steroids and cyclophosphamide. (**C**) Computed tomography of the abdomen performed on day 29 before treatment, showing ascites (arrows) that remained after treatment with steroids and cyclophosphamide. However, the severe edematous changes in the small intestine were slightly improved relative to day 9.

On admission, the patient had a body temperature of 36.9°C, a pulse of 109/min with sinus rhythm, and blood pressure of 133/78 mm Hg. Physical examination revealed pretibial pitting edema and diffuse small palpable purpuras on both lower extremities (Figure 1A, B) and on the lower abdomen. The patient’s heart and lung sounds were normal. His abdomen was slightly distended, but neither muscular guarding nor tenderness was detected. On auscultation, the bowel was hypoactive. There was no hepatosplenomegaly or existing masses. A neurological examination revealed no abnormalities. An electrocardiogram and X-rays of the chest and abdomen were essentially normal. Computed tomography of the patient’s abdomen demonstrated diffuse thickening of the bowel wall, with target signs in the small intestine and ascites (Figure 2A). The white blood cell count was 14,260/μL, hemoglobin was 16.4 g/dL, and C-reactive protein was 22.36 mg/dL. There was microscopic hematuria and proteinuria. A skin biopsy taken from the right leg showed purpura.

**Figure 3:**
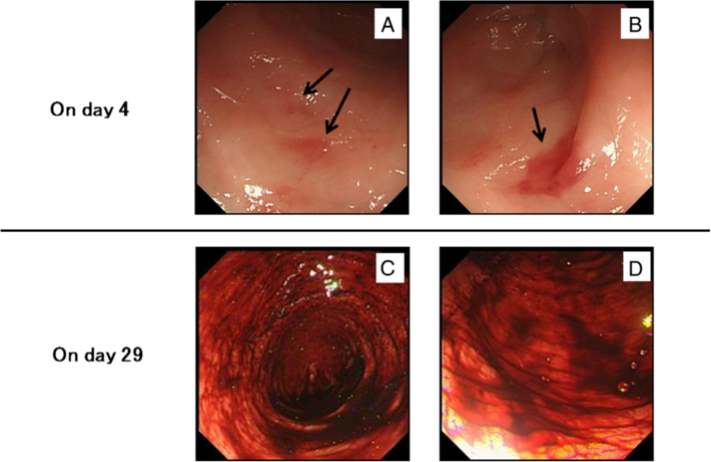
**Colonoscopy findings. **Colonoscopy images showing patchy erythema in the sigmoid colon (**A**) and rectum (**B**) on day 4, before treatment began. (**C**, **D**) Colonoscopy images showing fresh bleeding in the ascending sigmoid colon on day 29, after treatment with steroids and cyclophosphamide.

On the second hospital day, abdominal pain and melena in our patient appeared after the purpura improved and before treatment with steroids or immunosuppressants. Upper and lower gastrointestinal endoscopic investigation on day 4 revealed irregular ulcerations in the stomach and the duodenum and erythema in the sigmoid colon. The ascending colon and cecum were not observable, owing to the presence of a stricture caused by severe edema of the mucosa. However, there were patchy areas of erythema in the sigmoid colon (Figure 3A) and rectum (Figure 3B). Cutaneous leukocytoclastic vasculitis was confirmed by skin biopsy. The patient was diagnosed with HSP on the basis of American College of Rheumatology criteria [10]. Computed tomography of the abdomen performed on day 9 showed severe edematous and dilatational changes in the small intestine and ascites before treatment with steroids and cyclophosphamide (Figure 2B).

**Figure 4:**
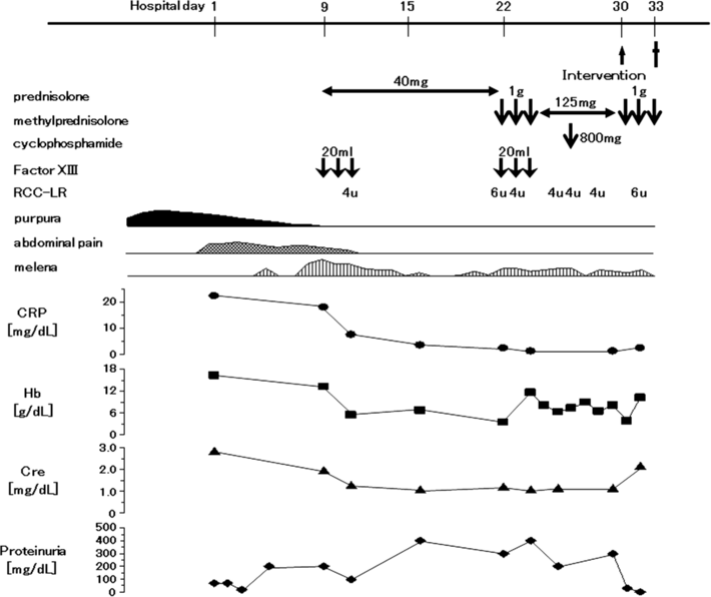
**Treatment and clinical course. **Top panel, doses and schedules of drug therapies. Middle panels, clinical findings. Abdominal pain and melena appeared after improvement of purpura and before treatment with steroid and immunosuppressants. The patient’s melena and proteinuria did not improve after treatment with steroids, cyclophosphamide, infusion of RCC-LR (red cell concentrates – leukocytes reduced), and intravenous PGE1 (prostaglandin E1). Bottom panels, laboratory findings. Cre, creatinine; CRP, C-reactive protein; Hb, hemoglobin.

**Figure 5:**
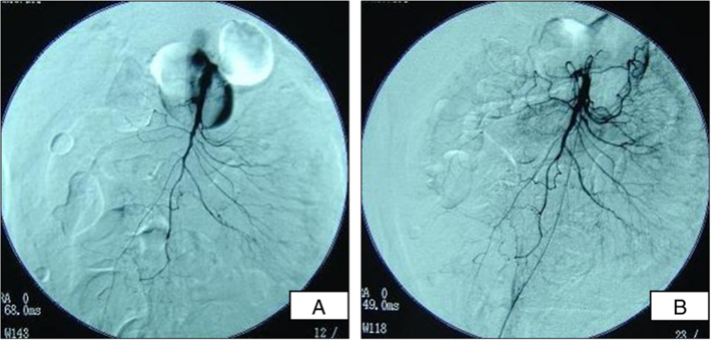
**Supramesenteric artery angiography. **(**A**) Mesenteric angiogram showing absence of bleeding points and vascular occlusion, but presence of narrowing and irregularity of major branches of the superior mesenteric artery. (**B**) Mesenteric angiogram taken on day 30, showing blood flow and reduced vascular narrowing after catheter injection of 10 μg of PGE1 and 20 mg of prednisolone sodium succinate into the superior mesenteric artery.

After intravenous treatment with Factor XIII concentrate (20 mL/day for 3 consecutive days) and prednisolone sodium succinate (40 mg/day for 13 consecutive days; see Figure 4 for dosage schedules of all drugs), the skin lesions and abdominal pain disappeared completely. However, gastrointestinal hemorrhage and mild proteinuria remained (Figure 4) and did not improve upon treatment with intravenous methylprednisolone (pulse dose of 1000 mg/day for 3 consecutive days) and intravenous cyclophosphamide (800 mg on 1 day). Twenty-nine days after treatment began, the ascites remained, and there were severe edematous changes in the small intestine (Figure 2C). Colonoscopy confirmed frank bleeding from the ascending sigmoid colon (Figure 3C, Dc, d). Neither bleeding points nor vascular occlusion was detected by mesenteric angiography. However, angiography revealed narrowing and irregularities in the major branches of the superior mesenteric artery and absence of blood flow, indicative of NOMI (Figure 5Aa). On day 30, blood flow in the superior mesenteric artery increased and vascular narrowing improved after catheter injection of 10 μg of PGE1 and 20 mg of prednisolone sodium succinate into the artery (Figure 5B). After the intervention, proteinuria decreased. However, subsequent continuous intra-arterial infusion of papaverine hydrochloride (100 mg/h) did not improve the patient’s gastrointestinal hemorrhaging, and renal function deteriorated. He died on the 33rd hospital day.

**Figure 6:**
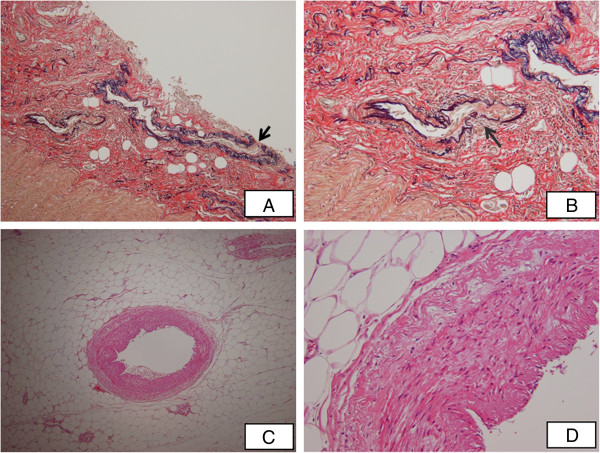
**Autopsy findings. **Biopsy samples were fixed and stained with hematoxylin and eosin. Histopathology of the jejunal submucosa shows (**A**) an exposed small vessel (arrow) (x40) and (**B**) exposed vessel and ruptured elastic laminae in the tunica externa of that vessel (x100). Histopathology of the tunica media of the superior mesenteric artery shows (**C**) no active vasculitis or thrombosis at autopsy (x40), but (**D**) edematous change (x200).

Examination at autopsy showed widely distributed jejunoileal ulcers of irregular form. Histopathologic examination of the submucosa of the jejunum revealed an exposed small vessel (Figure 6A), as well as obliteration of small vessels and ruptured elastic laminae in the tunica externa of those vessels (Figure 6B). There was also an abundance of fibroblasts under the submucosa of the jejunum but no regeneration of the epithelium. There was no evidence of active vasculitis or thrombosis in the tunica media of the superior mesenteric artery (Figure 6C), although there were edematous changes (Figure 6D). These findings suggest that severe vasospasm due to small-vessel vasculitis before treatment with PGE1 plus steroid had caused wide ischemic changes and delayed regeneration of the epithelium in the submucosa of the small intestine. We considered that the primary cause of death was bleeding from an exposed small vessel, without apparent active vasculitis in the jejunal submucosa. Other findings were typical of HSP and included mesangial proliferative glomerulonephritis with crescent formation.

## Discussion

Although HSP is typically a disease of children, adult cases can occur and present as more severe disease. Serious complications related to gastrointestinal involvement include intussusception, infarction, and perforation [7,11,12]. HSP patients who are older than 60 years at onset and who have renal or gastrointestinal involvement have a poor prognosis [5-8,13]. Some patients with gastrointestinal involvement die despite treatment with steroids and immunosuppressants [8,13]. Among adult patients with HSP, 24.1% have initial onset with gastrointestinal involvement before the cutaneous rash [11], and those manifestations may be helpful for early diagnosis and for selecting appropriate management strategies [12].

Few reports of NOMI associated with small-vessel vasculitis have been published. There has been one case report of vasculitic lesions that resulted in dialysis-related hypotension and NOMI, but this was associated with giant cell arteritis [14]. NOMI in our patient might have been caused by active vasculitis, but it appeared not to have been caused by hypotension or hypovolemia, and the patient did not receive drugs such as α-adrenergic agonists, digoxin, or β-receptor blocking agents, which are known to precipitate this condition [9]. NOMI has a high mortality rate, and early diagnosis and treatment are important for improving survival [15]. Early treatment of NOMI patients with continuous intravenous PGE1 increases survival rates [15,16]. Abdominal pain and melena in our patient appeared after the purpura improved and before treatment with steroids or immunosuppressants. The patient’s melena and renal dysfunction did not improve after treatment with steroids, cyclophosphamide, and intravenous PGE1 and steroids. At autopsy, an exposed small vessel was identified, but there was no evidence of active vasculitis in the submucosa of the jejunum. We suspect that the treatment for NOMI improved vasodilatation in the submucosa of the small intestine, thus improving the distribution of the locally injected steroid or the ongoing systemic steroid and immunosupressant to the submucosa of the jejunum. We considered that exposure of the small vessel, possibly as a result of the remodeling process in the submucosa, was the cause of death.

## Conclusion

Treatment of NOMI results in vasodilatation, thus resulting in more effective treatment of the active small-vessel vasculitis associated with HSP.

## Consent

Written informed consent was obtained from the kin of the patient for publication of this Case Report and any accompanying images. A copy of the written consent is available for review by the Editor-in-Chief of this journal.

## Abbreviations

Cre: Creatinine; CRP: C-reactive protein; Hb: Hemoglobin; HSP: Henoch-Schönlein purpura; NOMI: Non-occlusive mesenteric ischemia; PGE1: Prostaglandin E1; RCC-LR: Red cell concentrates – leukocytes reduced.

## Competing interests

We declare no non-financial competing interests.

None of the authors received grants for this study.

## Authors’ contributions

CO examined the patient and contributed to manuscript preparation. NT examined the patient, took part in discussions about the patient, and was involved in manuscript preparation and editing. MT and TO implemented treatments and performed diagnostic imaging. SS performed endoscopy of the colon. T Tsuburai was involved in discussions regarding the patient. HM performed the autopsy and diagnostic pathology. T Takemura contributed to discussions and pathological diagnosis. KA contributed to discussions regarding the patient. All authors read and approved the final manuscript.
